# A randomised Phase II/III study to evaluate the efficacy and safety of orally administered *Oxalobacter formigenes* to treat primary hyperoxaluria

**DOI:** 10.1007/s00240-017-0998-6

**Published:** 2017-07-17

**Authors:** Dawn Milliner, Bernd Hoppe, Jaap Groothoff

**Affiliations:** 1 Division of Nephrology, Departments of Pediatrics and Internal Medicine, Mayo Clinic, Rochester, MN USA; 2 Division of Pediatric Nephrology, University Children’s Hospital, Bonn, Germany; 3 Academic Medical Center, University of Amsterdam, Amsterdam, The Netherlands

**Keywords:** Primary hyperoxaluria, Oxalobacter formigenes, Oxalate, Kidney, Kidney function

## Abstract

Primary hyperoxaluria (PH) patients overproduce oxalate because of rare genetic errors in glyoxylate metabolism. Recurrent urolithiasis and/or progressive nephrocalcinosis are PH hallmarks and can lead to kidney damage, systemic oxalosis and death. Based on previous studies, we hypothesised that treatment with the oxalate-metabolizing bacterium *Oxalobacter formigenes* would mediate active elimination of oxalate from the plasma to the intestine of PH patients, thereby reducing urinary oxalate excretion (Uox). The efficacy and safety of *O. formigenes* (Oxabact™ OC3) were evaluated for 24 weeks in a randomised, placebo-controlled, double-blind study. The primary endpoint was reduction in Uox. Secondary endpoints included change in plasma oxalate (Pox) concentration, frequency of stone events, number of responders, and Uox in several subgroups. Additional post hoc analyses were conducted. Thirty-six patients were randomised; two patients withdrew from placebo treatment. Both OC3 and placebo groups demonstrated a decrease in Uox/urinary creatinine ratio, but the difference was not statistically significant. No differences were observed with respect to change in Pox concentration, stone events, responders’ number or safety measures. In patients with estimated glomerular filtration rate (eGFR) < 90 mL/min/1.73 m^2^, Pox increased by 3.25 µmol/L in the placebo group and decreased by −1.7 µmol/L in the OC3 group (*p* = 0.13). After 24 weeks, eGFR had declined to a greater degree in the placebo than in the OC3 group: −8.00 ± 2.16 versus −2.71 ± 2.50; *p* = 0.01. OC3 treatment did not reduce urinary oxalate over 24 weeks of treatment compared with placebo in patients with PH. The treatment was well tolerated.

**Electronic supplementary material:**

The online version of this article (doi:10.1007/s00240-017-0998-6) contains supplementary material, which is available to authorized users.

## Introduction

Primary hyperoxaluria (PH) is a rare condition arising from autosomal recessive inborn errors in glyoxylate metabolism that lead to endogenous overproduction of oxalate. In humans, oxalate cannot be degraded and thus must be eliminated by the intestine and the kidneys. To avoid hyperoxalemia, animal experiments and experiments in human intestinal cell lines in vitro indicate that the body excretes oxalate through active transport from plasma to the gut [1–3] and transports oxalate in the renal tubuli by both passive osmotic and active processes [4]. Under physiological conditions, most oxalate is excreted by the kidneys; when kidney function is compromised, gut excretion appears to play a greater role [5, 6].

In plasma, oxalate begins to crystallise with calcium once the concentration of free, unbound oxalate reaches the critical saturation point of approximately 30–50 μmol/L [7, 8]. Although the concentration of oxalate in plasma never exceeds this concentration in healthy individuals, inborn errors in metabolism cause patients with PH to produce 0.7–7 mmol oxalate per day [9, 10]. Consequently, when kidney function is compromised, plasma concentrations increase and calcium oxalate can be deposited not only in kidneys, but also in blood vessel walls, bones, as well as other organs and tissues throughout the body [10].

Renal tubule epithelial and parenchymal cells may be damaged by high concentrations of oxalate in the glomerular filtrate and by calcium oxalate crystals. The crystals in particular can result in parenchymal inflammation, interstitial fibrosis and, consequently, in progressive decline of kidney function [11, 12]. In the most severe form of primary hyperoxaluria (type 1), this process eventually results in end-stage renal disease (ESRD) [10, 13]. The median age for onset of ESRD in type 1 PH is 24–33 years [13–15]. A recently published univariate analysis showed that the most important parameters governing the progression to ESRD in PH patients were type of PH, baseline urinary oxalate excretion by quartile, and estimated glomerular filtration rate (eGFR) at diagnosis [16]. During follow-up, only oxalate excretion more than 1.87 mmol/1.73 m^2^/24 h (quartile 4) was significantly associated with progression to ESRD. Conservative treatment includes high fluid intake, oral crystal inhibitors (citrate, magnesium or phosphate) [10, 17, 18], and pyridoxine treatment for some patients with PH type 1 [17]. Combined liver and kidney transplantation is the only curative therapy for PH type 1, although this is associated with its own challenges [9].

*Oxalobacter formigenes* is a strictly anaerobic, commensal bacterium that uses oxalate as its sole carbon source [20–22] and has been suggested as a potential treatment for PH. In animal models, *O. formigenes* mediates enteric elimination of oxalate by activating transport of oxalate from the plasma into the intestine [23, 24]. Enteric elimination of oxalate can occur by passive flow of oxalate across the gut epithelium in response to a concentration gradient, as well as by active flux mediated by specific transporter proteins [1, 23, 24]. This process facilitates removal of oxalate from plasma and elimination in faeces, thereby potentially reducing the endogenous oxalate burden.

In a previous open-label study, patients with PH received enteric-coated capsules of lyophilised *O. formigenes* genotype 1 (strain HC-1) administered orally for 4 weeks [25]. Reduction in urinary oxalate excretion of 38–92% was observed in four out of six patients with normal kidney function. However, this reduction was not confirmed in a subsequent larger study (OC3-DB-01) that used a modified formulation (OC3b) administered for 24 weeks [26].

In the current study, patients with PH were treated for 24 weeks. The aim of the study was to evaluate the efficacy and safety of a larger dose of the new OC3 formulation.

## Methods

### Study design

The OC3-DB-02 study (ClinTrials.gov Identifier: NCT01037231) was conducted at centres (one in each country) in Germany, the Netherlands and the USA between January 2010 and January 2011. The study was randomised, placebo-controlled and double-blind.

Patients performed two 24-hour urine collections during screening, which took place up to 6 weeks before randomisation (week 0). Randomisation was strictly consecutive and took place after each patient’s eligibility was confirmed. Within each geographic region (European Union and USA), patients were randomised in a 1:1 ratio. Randomisation was stratified by estimated renal function (above or below 90 mL/min/1.73 m^2^), as defined by the patient’s eGFR at screening. Glomerular filtration rate was estimated by the Schwarz equation in subjects <18 years of age (*n* = 25) and MDRD in those ≥18 years of age (*n* = 11). Patients entering the study received OC3 or placebo twice daily for 24 weeks. The treatment was provided as a powder, which was dissolved in a buffer that was developed to sustain the survival of *O. formigenes* cells under simulated gastric and intestinal conditions. Patients were provided with sachets of OC3 (or placebo) and sachets of buffer powder. One sachet of OC3 (or placebo) powder was mixed with one sachet of buffer and reconstituted with 100–150 mL of water. The resulting suspension was administered orally. Dosing took place on a fasted stomach, 30–60 min before a meal. A 500-mg dose of OC3 consisted of one sachet of lyophilised *O. formigenes* strain HC-1 (not less than 10^7^ colony forming units (CFU)/dose). Each *O. formigenes* dose had the capacity to metabolise approximately 30 mmol (2640 mg) oxalate at 19 h. The sachet of buffer contained sodium bicarbonate (1.5 g) and citric acid (0.5 g). The placebo sachet was visually identical to the OC3 sachet and contained inert excipients. The sachets were kept in a refrigerator in the patient´s home. Because *O. formigenes* cells are sensitive to low pH, patients were also instructed to take a once-daily dose of a delayed-release oral formulation of a proton pump inhibitor (esomeprazole) to increase the pH in the stomach. This medication was provided as a powder in sachets; patients 11 years of age and older took 20 mg (two sachets) of esomeprazole per day, whereas patients younger than 11 years old took 10 mg of esomeprazole per day. The dose was dissolved in water, according to instructions in the product information sheet.

During the treatment period, two 24-h urine samples were collected at week 8, week 16 and week 24. Urine collections were performed at the subject’s home. Samples were acidified at collection and transported to the clinical site. At the clinical site, the sample was checked against quality criteria (pH < 3 and collection time within 22–26 h), mixed and acidified to a pH of below 1.5 and divided into five aliquots of 50 mL each. Four of these were shipped in ambient temperature to the central lab (Mayo Clinic; Rochester, MN, USA) and one aliquot was stored at −20 °C at the clinical site.

The urine samples were ideally collected on two consecutive days and were required within 2 weeks prior to the planned clinic visit. Results from the analysis of the 24-h urine collections were monitored against urine acceptance criteria by an independent urine data review committee. The urine acceptance criteria were as follows: creatinine excretion within 10–35 mg/kg, collection time 22–26 h, less than 15% deviation in creatinine excretion (mg/24 h) from the mean level of two complete urine collections at baseline, or less than 20% deviation in creatinine excretion (mg/24 h) between the mean baseline level and each urine collection during follow-up. Samples with nonsensical values of creatinine or oxalate (unrealistic values suspected to be data entry errors or calculation errors) were not accepted. Any 24-h urine collection that did not meet the urine acceptance criteria at baseline or at week 24 was repeated once. If a repeat collection was needed at week 24, the patient was instructed to continue treatment until the repeat collection was completed (i.e., up to week 28 at the latest).

Clinic visits took place at week 8 and week 24; patients were contacted by telephone at monthly intervals between the visits. There was a post-treatment follow-up 4 weeks after the end of the treatment period, conducted by clinic visit (Germany) or a telephone call (Netherlands, USA).

### Study population

Patients were of either gender, aged 2 years or above and had a diagnosis of PH that was made using standard methods (analysis of urinary excretion levels of oxalate, glycolate and, in addition, either liver biopsy and/or genetic testing). Eligible patients had a urinary oxalate excretion equal to or more than 1.0 mmol/24 h/1.73 m^2^, based on the mean value of urine collections during baseline. Patients were also required to have an estimated glomerular filtration rate (eGFR) more than 40 mL/min/1.73 m^2^ or a creatinine clearance equal to or more than 40 mL/min/1.73 m^2^. The creatinine clearance was an approximation, since serum sampling and urine collection did not coincide.

If patients were receiving vitamin B6 (pyridoxine), the dose needed to have been stable for at least 3 months before screening and they had to agree to maintain the same dose for the duration of the study. Patients were not able to start vitamin B6 treatment once they had entered the study.

### Efficacy assessment

The primary endpoint was percentage change in urinary oxalate (expressed as molar oxalate to creatinine ratio) from baseline to week 24. The mean of the two collections during baseline was used. Urinary oxalate excretion was measured using an enzymatic assay at the renal laboratory of the Mayo Clinic (modified from Kasidas and Rose [27]). Urinary creatinine was also measured in the 24-h sample with a standard enzymatic method at the renal laboratory of the Mayo Clinic. The method was based on the enzymatic conversion of creatinine to creatine and then to sarcosine by creatininase, followed by oxidation of sarcosine by sarcosine oxidase. The hydrogen peroxide produced was measured via a modified Trinder reaction using a colorimetric indicator (Roche diagnostics, IN. 2004).

Total plasma oxalate concentration (free oxalate plus protein-associated oxalate) was measured at the Genetic and Metabolic Disease laboratory of the Academic Medical Centre (Amsterdam, Netherlands) using gas chromatography-mass spectrometry [28]; the limit of quantification for this method was 0.5 µmol/L.

Stone events were defined as stone passage, stone removal, renal colic, macroscopic haematuria, as well as any increase in number or size of stones as defined from renal imaging. Stone events were detected by reporting of symptoms or observed by renal ultrasound at week 24.

Creatinine clearance and eGFR (calculated using the Schwarz formula in children [29] and the Modification of Diet in Renal Disease study equation in adults [30]) were used to assess the stability of kidney function over time.

The 24-h urine collections were analysed for sodium, potassium and urea nitrogen, as indicators of any significant variation in diet. In addition, calcium, citrate, magnesium and urine volume were measured.

### Safety assessments

Following initiation of treatment, the Investigator questioned all patients about adverse events (AEs) at clinic visits and during telephone calls. Signs and symptoms and reports by patients could also result in the identification of AEs. All AEs were recorded by the Investigator who coded them using the Medical Dictionary for Regulatory Activities Version 16.1 (MedDRA). Clinical laboratory measurements and vital signs were also assessed. Safety laboratory testing included the following: haematology [complete blood count (CBC) with differential and platelet count]; chemistry [blood urea nitrogen (BUN), creatinine, electrolytes (Na^+^, K^+^, Mg^++^, Ca^++^, HCO_3_, Cl), albumin, alkaline phosphatase, alanine aminotransferase (ALT), aspartate aminotransferase (AST), total bilirubin and total protein]; random urine for urine analysis (protein, glucose, pH).

### Statistical methods

The sample size estimations for this study were based on the post hoc results from the previous Phase II/III study (OC3-DB-01), where the standard deviation (SD) of the percentage change in molar oxalate to creatinine ratio from baseline to week 24 was 23%, ranging from 20 to 25% in subgroup analyses. The primary endpoint was evaluated using a Wilcoxon-Mann–Whitney test. The active treatment group was compared with placebo using a 1:1 randomisation and a two-sided significance level of 0.05, assuming normal distributions in the two groups with a mean difference of 30%. Thus, the sample size required to obtain a power of at least 90% was found to be 29.

An interim futility analysis of the primary efficacy endpoint was performed by an independent data monitoring committee (DMC) on data from 16 patients at week 8. The rationale was to have at least 50% of patients analysed and to do this before 50% of total study duration (in patient weeks) had elapsed. A conservative cut-off for conditional power of 20% was chosen, due to the choice of an intermediate endpoint and because of the small sample size. The recommendation from the DMC was that it would “probably be futile to continue the study to conclusion for efficacy reasons. The safety data should, however, not prevent a continuation of the study”. Following a separate review of the efficacy data by the Sponsor statistician, the Sponsor concurred with the DMC interpretation, but also noted a higher than anticipated intra-subject variability in the study’s primary efficacy endpoint (urinary oxalate), despite well-controlled urinary samples. Based upon these findings, the Sponsor and the three principal investigators agreed that, despite the recommendation from the DMC, the study should continue until conclusion. The reasoning for continuing the study was that no safety concerns had been identified, and, with the high variability in the primary efficacy endpoint, it was premature to draw conclusions on efficacy with the small amount of data available at that point in time. The study was more than two thirds complete by then, and there was significant value in obtaining the remaining data.

All endpoints based on percentage change from baseline measurements were analysed using the same methods as the primary analysis. A two-sided Fisher’s exact test was used to compare the percentage of responding patients (urinary oxalate to creatinine ratio decreased by more than 20% from baseline to week 24) in each treatment group. Frequency of stone events per treatment group was described as categorical data. Correlations were analysed using Spearman rank correlations within each treatment group.

Safety analyses were based on all randomised patients who received at least one dose of OC3 or placebo. Efficacy analyses were based on all randomised patients who received at least one dose of OC3/placebo and provided at least one post-baseline measurement of urinary oxalate.

## Results

### Study population

**Fig. 1:**
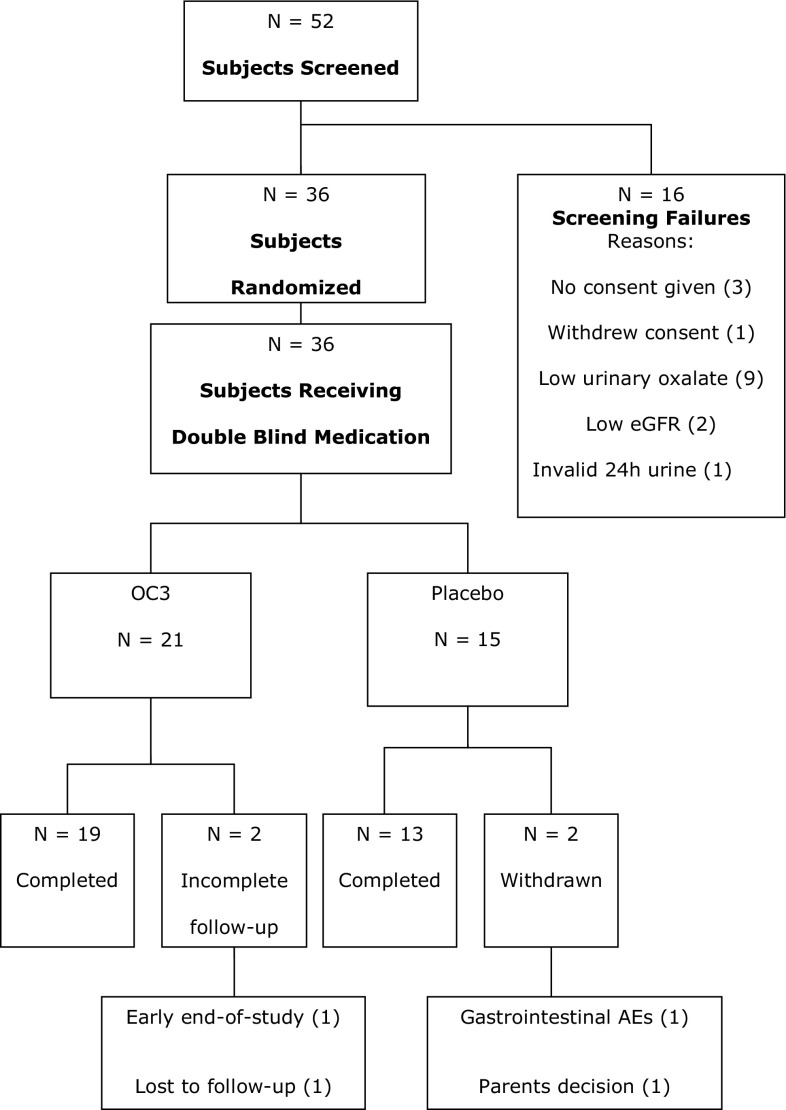
Study flowchart

Fifty-two patients were screened and 36 were randomised. The main reason for screening failure was urinary oxalate excretion below 1 mmol/24 h/1.73 m^2^ (Fig. 1). Thirty-two patients completed the study (19 receiving OC3 and 13 receiving placebo). In the OC3 group, two patients did not complete the study procedures as planned; in the placebo group, one patient withdrew due to gastrointestinal AEs and one patient was withdrawn by his parents after experiencing several AEs. A summary of patient characteristics of the study population is presented in Table 1.

**Table 1:** Baseline characteristics of study population

	OC3 (*N* = 21)	Placebo (*N* = 15)	Total (*N* = 36)
Age at screening, years			
*n*	21	15	36
Mean (SD)	16.2 (12.72)	22.6 (16.55)	18.8 (14.57)
Median	12.6	17.1	15.0
Min, Max	4, 54	7, 63	15, 63
*p* value^a^	0.10		
Age distribution at screening, *n* (%)			
Children (<12 years)	10 (47.6)	3 (20.0)	13 (36.1)
Adolescents (≥12, <18 years)	6 (28.6)	6 (40.0)	12 (33.3)
Adults (≥18 years)	5 (23.8)	6 (40.0)	11 (30.6)
*p* value^a^	0.117		
Ethnicity, *n* (%)			
Caucasian *n* (%)	20 (95.2)	15 (100)	35 (97.2)
Other *n* (%)	1 (4.8)	0 (0)	1 (2.8)
*p* value^a^	0.40		
Sex, *n* (%)			
Male	10 (47.6)	9 (60.0)	19 (52.8)
Female	11 (52.4)	6 (40.0)	17 (47.2)
*p* value^a^	0.47		
Primary hyperoxaluria type, *n* (%)			
Type I	18 (85.7)	13 (86.7)	31 (86.1)
Type II	3 (14.3)	2 (13.3)	5 (13.9)
*p* value^a^	0.936		
Time since diagnosis, years^b^			
*n*	21	14	35
Mean (SD)	5.50 (6.08)	15.44 (11.96)	9.47 (10.04)
Median	3.30	13.10	5.70
Min, Max	−0.1^b^–23.1	4.8–41.6	−0.1–41.6
*p* value^a^	<0.001		
Age at diagnosis, years			
*n*	21	14	35
Mean (SD)	10.7 (12.98)	8.1 (11.51)	9.7 (12.31)
Median	5.0	5.0	5.0
Min, Max	0–53	2–47	0–53
*p* value^a^	0.56		
Frequency of stone events during past year, *n* (%)			
0 event	16 (76.2)	8 (53.3)	24 (66.7)
1 event	4 (19.0)	2 (13.3)	6 (16.7)
2 event	0 (0.0)	4 (26.7)	4 (11.1)
≥3 event	1 (4.8)	1 (6.7)	2 (5.6)
*p* value^a^	0.08		
Stone events during past three years			
*n*	21	15	36
Mean (SD)	2.0 (3.53)	1.7 (2.05)	1.9 (2.97)
Median	1.0	1.0	1.0
Min, Max	0–16	0–7	0–16
*p* value^a^	0.92		
Estimated glomerular filtration rate, mL/min/1.73 m^2^			
*n*	21	15	36
Mean (SD)	111.6 (48.60)	109.6 (40.26)	110.8 (44.71)
Median	121.0	103.0	107.0
Min, Max	36–188	44–203	36–203
*p* value^a^	0.822		
Renal function during the past year, *n* (%)			
Stable	20 (95.2)	15 (100)	35 (97.2)
Worsening	1 (4.8)	0 (0.0)	1 (2.8)
*p* value^a^	0.40		
CKD stage, *n* (%)			
Stage I (eGFR ≥ 90)	14 (66.7)	11 (73.3)	25 (69.4)
Stage II (eGFR ≥ 60, <90)	3 (14.3)	3 (20.0)	6 (16.7)
Stage III (eGFR ≥ 30, <60)	4 (19.0)	1 (6.7)	5 (13.9)
*p* value^a^	0.44		
Urinary oxalate excretion (mmol/24 h/1.73 m^2^) at baseline			
*n*	19	12	–
Mean (SD)	1.88 (0.50)	1.62 (0.52)	–
Median	1.74	1.56	–
Min, Max	1.1–2.8	1.1–2.5	–
*p* value^a^	0.32		

*eGFR* estimated glomerular filtration rate, *SD* standard deviation

^a^ *p* value obtained in post hoc analyses

^b^ Time since diagnosis was the date of screening minus the date of diagnosis; one patient was diagnosed after screening and hence, there was one negative value

Both groups were similar with respect to sex, ethnic origin and mean age, although there were more children in the OC3 group (48% aged below 12 years versus 20% in the placebo group). The mean time since diagnosis was shorter in the OC3 group (5.50 ± 6.08 years vs. 15.44 ± 11.96 years). The number of patients who had not had any stone events in the past year was higher in the OC3 group than in placebo (76 vs. 53%). Mean eGFR at screening was similar between the two groups (OC3: 111.6 mL/min/1.73 m^2^; placebo: 109.6 mL/min/1.73 m^2^), although a slightly higher percentage of patients in the OC3 group had entered Stage II or III chronic kidney disease than placebo (33 vs. 27% in placebo).

There were no clinically relevant differences between the OC3 and placebo groups with respect to PH disease history. The most frequently reported ongoing medical history events were renal and urinary disorders, with nephrocalcinosis being the most frequent one (OC3: 71% of patients; placebo: 73% of patients). With respect to frequency of renal and urinary disorders, there was no difference between the OC3 and placebo groups. The most frequently used prior medications were pyridoxine (vitamin B6), which was used by 61% of patients, followed by preparations containing citrate (potassium citrate and/or sodium citrate) used by 31% of patients.

### Urine collections

A total of 287 24-hour urine samples were collected during the study.

Fifty-six (20%) of these were not valid for analysis. Ten samples were not properly acidified and, therefore, discarded by the local laboratory; in two samples, there was extensive leakage and in two occasions, the daily collections were accidentally mixed at the laboratory. Of the 56 invalid urine samples, 21 were over-collected and 21 were under-collected, as judged by the creatinine content. During screening, six out of 36 subjects (17%) had to do repeat collections. At weeks 8, 16 and 24, about 40% of subjects had at least one sample that did not meet the urine acceptance criteria. The number of subjects with invalid urine samples due to creatinine variability was two (6%) at screening and 10 (29%) at each of the later time points.

### Primary endpoint

At baseline, the mean urinary oxalate per creatinine ratio was higher in the OC3 group (204.1 ± 72.39 mmol/mol) compared with the placebo group (138.1 ± 52.06 mmol/mol). In both groups, there was a decrease in the ratio of urinary oxalate excretion to creatinine excretion from baseline to week 24 (Table 2). The decrease was greater in the placebo group (−8.81% versus −4.50% in the OC3 group), but the difference between the groups did not achieve statistical significance (*p* = 0.22).

**Table 2:** Change in urinary oxalate to creatinine ratio from baseline

	Urinary oxalate per creatinine ratio (mmol/mol)			
	OC3		Placebo	
	Absolute values	% change to baseline	Absolute values	% change to baseline
Baseline				
*n*	20	–	12	–
Mean (SD)	204.05 (72.39)	–	138.08 (52.06)	–
Median	190.50	–	122.00	–
Min, Max	91.0, 415.0	–	80.0, 230.0	–
Week 8				
*n*	15	17^a^	9	10^a^
Mean (SD)	203.03 (93.26)	0.71 (21.29)	141.56 (58.88)	1.07 (25.47)
Median	179.0	−1.06	122.0	−2.74
Min, Max	119.0, 489.5	−30.1, 39.2	55.5, 224.0	−47.6, 37.8
Mean treatment difference	–	−0.36	–	–
95% confidence interval	–	−19.14, 18.42	–	–
*p* value^b^	–	0.98	–	–
Week 24				
*n*	15	20^a^	9	12^a^
Mean (SD)	194.4 (92.56)	−4.50 (17.90)	118.89 (65.26)	−8.81 (44.43)
Median	165.0	−8.01	86.0	−15.07
Min, Max	75.0, 451.0	−30.2, 33.8	49.0, 237.5	−64.5, 105.6
Mean treatment difference	–	4.31	–	–
95% confidence interval	–	−18.40, 27.01	–	–
*p* value^b^	–	0.22	–	–

*SD* standard deviation

^a^ Numbers were derived from a post hoc analysis after the finalisation of the Clinical Study Report (CSR)

^b^ *p* value was derived after a Wilcoxon-Mann–Whitney-test

### Secondary endpoints

#### Change in urinary oxalate excretion to creatinine excretion ratio from baseline to week 8

After eight weeks of treatment, there was no significant difference between the groups in urinary oxalate per creatinine ratio (*p* = 0.98). The OC3 group increased 0.71% and the placebo group increased 1.07% (Table 2).

#### Difference in number of stone events over 24-week treatment period

Nine patients (45%) in the OC3 group and seven patients in the placebo group (58%) experienced stone events during the 24-week treatment period. There was a similar number of events in each group (OC3: 12 events; placebo: 13 events).

#### Difference in number of responders

A patient was judged to have responded to treatment if the urinary oxalate to creatinine ratio decreased by more than 20% from baseline to week 24. There was a greater percentage of responders in the placebo group (five out of 12 patients; 42%) than in the OC3 group (five out of 20 patients; 25%), although the difference between the percentages did not achieve statistical significance (*p* = 0.44).

#### Change in plasma oxalate concentration from baseline to week 24

At baseline, the mean plasma oxalate levels were somewhat higher in the OC3 group (14.26 ± 7.62 µmol/L) than in the placebo group (9.83 ± 4.17 µmol/L). After 24 weeks of treatment, there was no significant difference between the groups; in the OC3 group, mean percentage change in plasma oxalate concentration over 24 weeks from baseline was a decrease by −12.20 ± 22.55% and in the placebo group an increase by 5.23 ± 68.53% (*p* = 0.75) (Table 3).

**Table 3:** Change in plasma oxalate concentration from baseline to week 24

	Plasma oxalate (μmol/L)			
	OC3		Placebo	
	Absolute values	% change to baseline	Absolute values	% change to baseline
Baseline^a^				
*n*	21	–	13	–
Mean (SD)	13.92 (7.578)	–	9.68 (4.024)	–
Median	11.00	–	8.90	–
Min, Max	7.3, 35.0	–	4.5, 18.5	–
Week 24^a^				
*n*	21	21	13	13
Mean (SD)	12.38 (7.79)	−12.20 (22.55)	9.82 (6.877)	5.23 (68.53)
Median	9.00	−14.42	6.70	−8.45
Min, Max	3.3, 35.0	−64.5, 30.3	3.0, 24.1	−60.3, 192.3
Mean treatment difference	–	−17.43	–	–
95% confidence interval	–	−50.21, 15.34	–	–
*p* value^b^	–	0.75	–	–

*SD* standard deviation

^a^ Numbers were derived from a post hoc analysis after the finalisation of the Clinical Study Report (CSR)

^b^ *p* value was derived after a Wilcoxon-Mann–Whitney-test

### Subgroup analyses

The primary endpoint was also evaluated in several subgroups of patients based on disease characteristics, age and region. No statistically significant differences were found in these analyses, apart from the subgroup of patients living in the USA where urinary oxalate per creatinine ratio was decreased by −2.02 ± 22.38% in the OC3 group and by −25.99 ± 11.17% in the placebo group; this difference achieved statistical significance in the efficacy population (*p* = 0.04).

### Post hoc analyses

#### Change from baseline in plasma oxalate

There was an inverse correlation between plasma oxalate and kidney function which was statistically significant at baseline (*r* = −0.77, *p* = 0.03).

The least squares mean change from baseline in plasma oxalate concentration after 24 weeks was negative in the OC3 group and positive in the placebo group (OC3: −1.54 ± 0.87 µmol/L; placebo: 0.14 ± 1.11 µmol/L), although not statistically significant (*p* = 0.24). In patients with eGFR below 90 mL/min/1.73 m^2^ (*n* = 11), the differences from screening to week 24 were greater (OC3: decrease by −1.71 ± 1.78 µmol/L; placebo: increase by 3.25 ± 2.36 µmol/L; *p* = 0.13). In patients with further reduced kidney function (eGFR less than 60 mL/min/1.73 m^2^; *n* = 5), the difference between the OC3 and placebo groups was greatest, though there was just one placebo subject in this group (OC3: −2.58 ± 0.72 µmol/L; placebo: 15.00 ± 1.45 µmol/L) (Supporting Table 1).

#### Change from baseline in eGFR

There was a statistically significant difference in change from baseline in eGFR in the group of patients with a baseline eGFR below 90 mL/min/1.73 m^2^ after 24 weeks of treatment. Both the OC3 group and the placebo groups declined in estimated kidney function over the study period, but the placebo group to a greater extent (OC3: −2.71 ± 2.50; placebo: −8.00 ± 2.16; *p* = 0.01).

#### Change in leucocytes

Calcium oxalate crystals can provoke parenchymal inflammation, interstitial fibrosis and nephrocalcinosis [11, 12, 31]. This crystal-induced damage in combination with renal obstruction can lead to progressive decline in kidney function. *Post hoc* analysis showed a statistically significant reduction of leukocytes in the OC3 group at the end of treatment (week 24) (−0.85 × 10^9^ (2.17) cells/L) compared with placebo (1.05 × 10^9^ (1.85) cells/L; (*p* = 0.01); this difference remained 4 weeks after treatment completion (*p* = 0.045). Absolute counts for leucocytes at the different time points are displayed in Supporting Table 2 and did not show statistically significant differences between groups.

### Safety and tolerability results

Treatment-emergent adverse events (TEAEs) were most frequently reported in the placebo group (63 events reported by 14 [93.3%] subjects) compared to the OC3 group (37 events reported by 15 [71.4%] subjects). The most frequently reported TEAEs were infections and infestations and gastrointestinal disorders. Nine gastrointestinal disorder events were reported by six subjects (29%) in the OC3 group and 19 events were reported by eight subjects (53%) in the placebo group (Table 4).

**Table 4:** Summary of adverse events reported by at least 10% of patients in either treatment group following initiation of treatment

	OC3 (*N* = 21)						Placebo (*N* = 15)						Total (*N* = 36)					
	Total		Not related		Possibly, Probably related		Total		Not related		Possibly, Probably related		Total		Not related		Possibly, Probably related	
	*n* (%)	*e*	*n* (%)	*e*	*n* (%)	*e*	*n* (%)	*e*	*n* (%)	*e*	*n* (%)	*e*	*n* (%)	*e*	*n* (%)	*e*	*n* (%)	*e*
Any adverse event	15 (71.4)	37	12 (57.1)	30	5 (23.8)	7	14 (93.3)	63	13 (86.7)	55	3 (20.0)	8	29 (80.6)	100	25 (69.4)	85	8 (22.2)	15
Gastrointestinal disorders	6 (28.8)	9	3 (14.3)	4	3 (14.3)	5	8 (53.3)	19	6 (40.0)	11	3 (20.0)	8	14 (38.9)	28	9 (25.0)	15	6(16.7)	13
Abdominal pain	1 (4.8)	1	1 (4.8)	1	0 (0.0)	0	3 (20.0)	4	2 (13.3)	2	1 (6.7)	2	4 (11.1)	5	3 (8.3)	3	1 (2.8)	2
Diarrhoea	2 (9.5)	2	1 (4.8)	1	1 (4.8)	1	2 (13.3)	2	1 (6.7)	1	1 (6.7)	1	4 (11.1)	4	2 (5.6)	2	2 (5.6)	2
Flatulence	2 (9.5)	2	0 (0.0)	0	2 (9.5)	2	3 (20.0)	4	1 (6.7)	1	2 (13.3)	3	5 (13.9)	6	1 (2.8)	1	4 (11.1)	5
Frequent bowel movements	0 (0.0)	0	0 (0.0)	0	0 (0.0)	0	2 (13.3)	2	0 (0.0)	0	2 (13.3)	2	2 (5.6)	2	0 (0.0)	0	2 (5.6)	2
Nausea	2 (9.5)	2	0 (0.0)	0	2 (9.5)	2	2 (13.3)	2	2 (13.3)	2	0 (0.0)	0	4 (11.1)	4	2 (5.6)	2	2 (5.6)	2
Vomiting	0 (0.0)	0	0 (0.0)	0	0 (0.0)	0	2 (13.3)	3	2 (13.3)	3	0 (0.0)	0	2 (5.6)	3	2 (5.6)	3	0 (0.0)	0
Infections and infestations	6 (28.6)	9	6 (28.6)	9	0 (0.0)	0	9 (60.0)	13	9 (60.0)	13	0 (0.0)	0	15 (41.7)	22	15 (41.7)	22	0 (0.0)	0
Nasopharyngitis	2 (9.5)	4	2 (9.5)	4	0 (0.0)	0	2 (13.3)	2	2 (13.3)	2	0 (0.0)	0	4 (11.1)	6	4 (11.1)	6	0 (0.0)	0
Upper respiratory tract infection	1 (4.8)	1	1 (4.8)	1	0 (0.0)	0	2 (13.3)	2	2 (13.3)	2	0 (0.0)	0	3 (8.3)	3	3 (8.3)	3	0 (0.0)	0
Injury, poisoning and procedural complications	1 (4.8)	1	1 (4.8)	1	0 (0.0)	0	2 (13.3)	2	2 (13.3)	2	0 (0.0)	0	3 (8.3)	3	3 (8.3)	3	0 (0.0)	0
Musculoskeletal and connective tissue disorders	4 (19.0)	4	4 (19.0)	4	0 (0.0)	0	3 (20.0)	3	3 (20.0)	3	0 (0.0)	0	7 (19.4)	7	7 (19.4)	7	0 (0.0)	0
Back pain	1 (4.8)	1	1 (4.8)	1	0 (0.0)	0	2 (13.3)	2	2 (13.3)	2	0 (0.0)	0	3 (8.3)	3	3 (8.3)	3	0 (0.0)	0
Psychiatric disorders	0 (0.0)	0	0 (0.0)	0	0 (0.0)	0	2 (13.3)	2	2 (13.3)	2	0 (0.0)	0	2 (5.6)	2	2 (5.6)	2	0 (0.0)	0
Renal and urinary disorders	4 (19.0)	6	4 (19.0)	6	0 (0.0)	0	7 (46.7)	13	7 (46.7)	13	0 (0.0)	0	11 (30.6)	19	11 (30.6)	19	0 (0.0)	0
Nephrolithiasis	2 (9.5)	3	2 (9.5)	3	0 (0.0)	0	4 (26.7)	6	4 (26.7)	6	0 (0.0)	0	6 (16.7)	9	6 (16.7)	9	0 (0.0)	0
Renal colic	0 (0.0)	0	0 (0.0)	0	0 (0.0)	0	2 (13.3)	6	2 (13.3)	6	0 (0.0)	0	2 (5.6)	6	2 (5.6)	6	0 (0.0)	0
Respiratory, thoracic and mediastinal disorders	1 (4.8)	1	1 (4.8)	1	0 (0.0)	0	2 (13.3)	2	2 (13.3)	2	0 (0.0)	0	3 (8.3)	3	3 (8.3)	3	0 (0.0)	0
Skin and subcutaneous tissue disorders	1 (4.8)	1	1 (4.8)	1	0 (0.0)	0	2 (13.3)	3	2 (13.3)	3	0 (0.0)	0	3 (8.3)	4	3 (8.3)	4	0 (0.0)	0
Acne	0 (0.0)	0	0 (0.0)	0	0 (0.0)	0	2 (13.3)	2	2 (13.3)	2	0 (0.0)	0	2 (5.6)	2	2 (5.6)	2	0 (0.0)	0
Vascular disorders	0 (0.0)	0	0 (0.0)	0	0 (0.0)	0	3 (20.0)	3	3 (20.0)	3	0 (0.0)	0	3 (8.3)	3	3 (8.3)	3	0 (0.0)	0
Hypertension	0 (0.0)	0	0 (0.0)	0	0 (0.0)	0	2 (13.3)	2	2 (13.3)	2	0 (0.0)	0	2 (5.6)	2	2 (5.6)	2	0 (0.0)	0

Adverse events are coded according to MedDRA version 16.1

Percentage is based on number of patients in safety analysis set

*n* number of patients, *e* number of events

Renal and urinary disorders (i.e., nephrolithiasis and renal colic) were also frequently reported; six events were reported by four subjects (19%) in the OC3 group and 13 events were reported by seven subjects (47%) in the placebo group. Of all gastrointestinal TEAEs, the vast majority of them were reported within the first 4 weeks after the start of study drug administration.

The majority of the TEAEs was of mild or moderate intensity and considered by the investigator to be unrelated to the OC3 treatment. In total, 85% of the reported TEAEs were considered not related, 15% as possibly/probably related and 0% as definitely related to the study medication (Table 4). In general, there were more TEAEs with higher severity in the placebo group. The majority of the events that were considered related to the OC3 treatment were gastrointestinal disorders, with five possibly related events reported in four subjects (19%). In the placebo group, eight possibly/probably related gastrointestinal events were reported in three subjects (20%).

One subject was withdrawn from the study due to TEAE (abdominal pain, flatulence and frequent bowel movements) after 3 weeks of treatment with placebo. Two subjects experienced serious adverse events (SAEs) which were unrelated to study medication: nephrolithiasis/urinary tract infection after 1 week with placebo, and nephrolithiasis after 23 weeks with placebo. There were no deaths. Adverse events reported by at least 10% of patients are shown in Table 4.

No significant safety trends were observed with respect to mean laboratory safety measurements, vital signs or physical examinations.

## Discussion

The oxalate kinetics in PH are complex and depend on the patient’s individual oxalate production, kidney function and relative levels of urinary oxalate excretion and plasma oxalate, as well as the presence or absence of systemic oxalate crystal deposition.

The results of the current study demonstrate that treatment with OC3 twice daily for 24 weeks was safe and well tolerated in patients with PH. The primary endpoint, change in urinary oxalate excretion per creatinine ratio from baseline to week 24, did not show benefit from OC3 treatment.

A recent study used OC5, a more potent formulation of *O. formigenes* with faster recovery time (time for the bacteria to recover their oxalate degrading capacity from the lyophilised state measured as viable cell count). This study revealed some unexpected effects of OC5 including an increased urinary excretion of oxalate and calcium; this suggested that systemic calcium oxalate deposits might be mobilised [32]. These findings, taken together with the important factors for progression to ESRD in PH patients as described by Zhao et al. [16], resulted in the decision to conduct post hoc analyses to investigate the same effects in the OC3-DB-02 study.

The post hoc analyses suggested a trend toward increased total plasma oxalate in the placebo group, while total plasma oxalate remained stable to reduced in the OC3-treated group. OC3 may enhance the secretion of oxalate from plasma to the small bowel, thus reducing the oxalate burden. However, the treatment time was too short to confirm these findings.

Further evaluation of the study drug formulation used in this study has revealed that the bacteria had a low viability and a slow recovery time, which might have influenced the treatment response in a negative way. After completion of this study, improvements in manufacturing methods and validation assays resulted in a new, more concentrated formulation, OC5 [32]. OC5 is currently undergoing further clinical trial evaluation.

Mechanisms of oxalate transport in the human gut are poorly understood. An alternative explanation of the lack of observed effect on urine oxalate excretion is that human intestinal epithelium is not responsive to *O. formigenes* effects on oxalate secretion, in contrast to what has been observed in animal models.

In conclusion, OC3 treatment was well tolerated, but was not found to reduce urinary oxalate excretion. *Post hoc* analyses, suggested possible effects of OC3 on plasma oxalate concentration levels, and leucocyte cell count that need to be further evaluated.

## Electronic supplementary material

Below is the link to the electronic supplementary material.

Supplementary material 1 (DOCX 40 kb)
