# Supplementary material for: A randomised Phase II/III study to evaluate the efficacy and safety of orally administered Oxalobacter formigenes to treat primary hyperoxaluria
Source: Urolithiasis. 2017 Jul 17;46(4):313–23. doi: 10.1007/s00240-017-0998-6 (PMC6061479; doi:10.1007/s00240-017-0998-6)
Supplement: Supplementary file 1 — Supplementary material 1 (DOCX 40 kb) [file 240_2017_998_MOESM1_ESM.docx]

A randomised Phase II/III study to evaluate the efficacy and safety of orally administered Oxalobacter formigenes to treat primary hyperoxaluria (Urolithiasis)

Authors: Dawn Milliner, Bernd Hoppe, Jaap Groothoff

Corresponding author: Dr Dawn S. Milliner, Division of Nephrology, Departments of Pediatrics and Internal Medicine, Mayo Clinic, Rochester, Minnesota, USA. Tel: +1 507-284-2511, E-mail: milliner.dawn@mayo.edu

**Supplementary data**

**Supporting Table 1**. Change from baseline in plasma oxalate (µmol/L) by week, treatment and baseline kidney function.

|  |  | **Change from baseline in plasma oxalate (µmol/L)** | | | | | | |
| --- | --- | --- | --- | --- | --- | --- | --- | --- |
| **Treatment week** |  | **Full population** | | **Baseline eGFR <90 mL/min/1.73 m^2^** | | | **Baseline eGFR <60 mL/min/1.73 m^2^** | |
|  |  | **OC3 (N=21)** | **Placebo (N=13)** | **OC3 (N=7)** | **Placebo (N=4)** | | **OC3 (N=4)** | **Placebo (N=1)** |
| Week 8 | LS Mean (SE) | 0.09 (0.799) | 1.57 (1.015) | 0.50 (1.717) | | 1.80 (2.272) | 1.28 (1.269) | 13.10 (2.538) |
|  | p-value | 0.259 | – | 0.659 | | – | 0.025 | – |
| Week 24 | LS Mean (SE) | -1.54 (0.869) | 0.14 (1.105) | -1.71 (1.784) | | 3.25 (2.361) | -2.58 (0.724) | 15.00 (1.448) |
|  | p-value | 0.241 | – | 0.128 | | – | – | – |
| eGFR=estimated glomerular filtration rate; LS=least squares; SE=standard error | | | | | | | | |

Supporting Table 2. Summary of leucocytes (10^9^/L) at Baseline, Week 8, Week 24 and Week 28.

| **Week** | **OC3**  **(N=21)** | **Placebo**  **(N=13)** | **p-value** |
| --- | --- | --- | --- |
| **Baseline** | | | |
| N | 21 | 13 |  |
| Mean (SD) | 6.94 (1.93) | 6.10 (1.23) | 0.376 |
| Median | 6.70 | 6.30 | – |
| Min, Max | 4.50, 12.00 | 3.70, 7.59 | – |
| **Week 8** | | | |
| N | 21 | 13 |  |
| Mean (SD) | 6.60 (1.54) | 6.21 (1.60) | 0.547 |
| Median | 6.43 | 6.10 | – |
| Min, Max | 4.76, 10.70 | 3.80, 9.22 | – |
| **Week 24** | | | |
| N | 21 | 13 |  |
| Mean (SD) | 6.09 (1.03) | 7.16 (2.46) | 0.172 |
| Median | 5.80 | 6.22 | – |
| Min, Max | 4.20, 8.20 | 4.40, 13.43 | – |
| **Week 28** | | | |
| N | 8 | 6 |  |
| Mean (SD) | 6.43 (0.93) | 7.45 (0.90) | 0.081 |
| Median | 6.31 | 7.46 | – |
| Min, Max | 5.09, 8.07 | 6.07, 8.68 | – |
| SD=Standard deviation | | | |
